# Mothers’ accounts of their stillbirth experiences and of their subsequent relationships with their living infant: an interpretative phenomenological analysis

**DOI:** 10.1186/s12884-015-0700-3

**Published:** 2015-10-13

**Authors:** A. Meltem Üstündağ – Budak, Michael Larkin, Gillian Harris, Jacqueline Blissett

**Affiliations:** The School of Psychology, Bahçeşehir University, Istanbul, Turkey; The School of Psychology, University of Birmingham, Birmingham, Edgbaston UK

**Keywords:** Stillbirth, Stillborn, Subsequent pregnancy following stillbirth, Parenting following stillbirth, Loss and grief, Continuing bonds, IPA, Interpretative phenomenological analysis

## Abstract

**Background:**

Due to contradictory findings regarding the effects of seeing and holding stillborn infants on women’s worsening mental health symptoms, there is a lack of clear of guidance in stillbirth bereavement care. Although some current research examines this phenomenon we are still not certain of the meaning of such experiences to women and what effects there may be on her subsequent parenting. Thus the present study focuses on the meaning of the stillbirth experience to women and its influence on the subsequent pregnancy and subsequent parenting from the mothers’ own experiences.

**Methods:**

A purposive sample of six women who experienced a stillbirth during their first pregnancy and who then went on to give birth to a living child after a further pregnancy, took part in email interviews, providing rich and detailed experiential narratives about both the stillbirth itself, and their relationship with their living child. An Interpretive Phenomenological Analysis was carried out in order to focus on mothers making sense of such experiences.

**Results:**

Analysis of written accounts led to the development of three overarching themes. In ‘Broken Canopy’, ‘How This Happened’ and ‘Continuing Bonds’, their accounts revealed an ongoing process where women accepted a new ‘unsafe’ view of the world, re-evaluated their view of self and others, and established relationships with both the deceased and the living infant.

**Conclusions:**

This study provided an insight into the stillbirth experience of mothers and its meaning to them with an existential focus. Typically the mother struggled with the contradictory process of accepting the existence of her deceased baby (this baby once lived) while being aware of the nonexistence (this baby). Meeting the dead baby was a crucial point at which the mother started processing her grief. The importance of individual differences in dealing with stressful situations was highlighted in terms of attachment strategies. Subsequent parenting experiences of mothers were very much influenced by their own previous experiences. Although some mothers managed to integrate this trauma into their life some remained very concerned and anxious about future and this anxiety then translated into their parenting experiences.

## Background

Stillbirth and its consequences have attracted some research interest. However, there still remains an uncertainty particularly about how to advise medical staff of the necessary steps that should be taken around stillbirth bereavement care. Before 1970s, preventing bereaved parents from seeing their stillborn infant was standard practice. In a landmark paper, Lewis outlined advice for health practitioners on how to facilitate the grief process by allowing parents to create memories with the deceased baby, such as naming the baby, and particularly encouraging women to see their stillborn babies [1]. It was suggested that seeing the dead baby was actually important for the grieving process. This approach was adopted as part of stillbirth bereavement care at the UK hospitals until the late nineties. However, a controversial study by Hughes et al. [2], suggested that women who did not see or hold their child had a lower prevalence of depression in their subsequent pregnancy. These women also exhibited fewer symptoms of anxiety and post-traumatic stress disorder (PTSD). The significance of this study was challenged by Brabin [3] on the grounds of inconclusive statistical differences and validity issues. Despite this, and without further empirical evidence, NICE (2007) guidance changed and the informed practice was not to encourage mothers to see or hold their stillborn infant [4]. Voluntary organisations (e.g. SANDS, UK) campaigned for the parents’ right to be offered to see or hold their baby, brought about change in the guidance, and subsequently, informed practice was to offer the options to parents to choose. These polarised views in dealing with stillborn care particularly for stillbirth bereavement care are still evident, however, in research informed guidance. Recently, in a review of the stillbirth and its management, it was suggested that parents need to be supported around approaching their stillborn baby [5]. Other recent findings found that, mothers who saw and held their stillborn babies, experienced lower levels of anxiety and depressive symptoms [6]. However during pregnancy subsequent to stillbirth, mothers appeared to have less depressive symptomology but more symptoms of anxiety if they had seen or held their stillborn babies. Similarly, an overall positive effect of having held a stillborn baby was found, particularly for births after 37 weeks gestation [7]. A recent study also found that mothers felt more comfortable and less frightened if the health care staff supported assumptive bonding by simply offering the baby to the mother, without emphasising the choice of whether she wanted to see or hold the baby [8]. This may be because the act of choosing implies that the mothers are doing something that they are not supposed to do. Furthermore Ryninks and colleagues [9] also highlighted that although contact with the stillborn infant caused distress to some women, they acknowledged the importance of such contact and proposed that this was the right decision to take. However, we still need to understand the individual circumstances surrounding the difficult decisions around stillbirth and use this to inform clinical guidance so that consistent reliable care is given to women.

The majority of studies used to inform stillbirth bereavement care are quantitative in nature. Recent qualitative studies have provided a closer look into the woman’s own experiences in relation to the care and support that she received from health practitioners [10], and the mother’s view on their interactions with the health care staff before and after stillbirth [11], as well as with their parenting experiences [12]. However, to date no other research with an existential focus has looked at what stillbirth actually means to women. This is an important aspect that is missing from the research literature, considering individual differences dealing with trauma [13]. Accounts of individuals will offer a useful insight into the meaning of stillbirth experience and the hard decisions that mothers have to make in that context.

Furthermore current research also suggests that mothers become anxious in their subsequent pregnancies and that this anxiety may have prolonged consequences for both mothers and the subsequent infant, for example increased psychopathology [14, 15] and disorganised infant attachment in subsequent infants [16].

Few studies have looked at the subsequent pregnancy and the ensuing relationship with the infant. One follow-up study drew attention to the mothers’ different perceptions and their attitudes toward the infants born subsequent to a stillbirth in comparison to their other children (e.g. ‘the vulnerable child’ or ‘the replacement child’) and called for further qualitative studies where a mother’s view of her subsequent child, and individual differences between mothers, could be understood and identified during stillbirth bereavement care [17]. Similarly, another recent qualitative study has also emphasized the impact of a loss of an infant on the subsequent parenting of mothers [18], and indicated a paradoxical pattern (trying to hold their subsequent child emotionally close, but aloof at the same time) in their parenting styles. However, this emerging area of research requires further investment in order to understand the factors that may be affecting a mother’s relationship with the subsequent infant, including an examination of parenting experiences.

Therefore, this study will focus on the meaning of the stillbirth experience to women and its influence on the subsequent pregnancy and subsequent parenting from the mothers’ own perspectives. The primary questions of this study are: How do women make sense of their experiences of stillbirth? How do mothers who have previously experienced a stillbirth make sense of their relationship with a subsequent infant? The secondary question of the study is: how do the mothers’ accounts of stillbirth and their relationship with subsequent children relate to the existential, cognitive (appraisal), and developmental (attachment) theories of psychology?

### Reflexivity

The first author experienced stillbirth 9 years ago and currently provides counselling services, particularly to women who have experienced prenatal and postnatal losses. She is a reliable Adult Attachment Interview, [19] coder and aware that she has pre-formed ideas around the stillbirth experience. These were noted during the analysis of each participant’s written accounts and during discussions which took place at supervision meetings with her supervisor. The second author is a phenomenological psychologist with an interest in the cultural context of personal experiences. The third author is a consultant clinical psychologist, interested in attachment issues. The fourth author is an applied developmental psychologist, whose specific interest lies in parenting and childhood.

## Methods

### Approach

A semi structured interview [20] was chosen to gain a detailed picture of accounts of mothers’ experiences. This interview was carried out by email to give participants time and space for remembering and reflecting on their own experiences without being overwhelmed. Written accounts were then analysed using interpretative phenomenological analysis (IPA) [21]. IPA consists of case by case analysis of a number of accounts in a particular context and provides in-depth understanding of the meaning of a shared experience. For this reason IPA requires purposive sampling of a homogenous and small group [21, 22]. Analysis focused first on how women make sense of their experiences of their stillborn babies and their relationship with their subsequent infants. A second focus was on how women’s account of their stillbirth experiences related to existential, cognitive (Appraisal) and developmental (Attachment Theory) components.

### Recruitment

After obtaining Ethical approval from the University of Birmingham for this study, the recruitment was carried out on internet-based social support websites – Facebook - Stillbirth group, Twitter - After Stillbirth, The Birth Trauma Association (BTA) and a US based pregnancy loss support forum- Share.

### Participants

A purposive sample of 6 women whose first pregnancy ended with a stillbirth and had since had a living infant (who was between the age of 4 months to 4 years old), took part in this study. The contextual details of the participants are presented in Table 1. One of the participants had a twin pregnancy which ended with a stillbirth and premature birth, followed by neonatal death. However, like other participants this pregnancy was her first pregnancy. Her stillbirth experiences and her relationship with her living daughter were shared in this research. The time gap between the stillbirths to subsequent live births varied from 15 to 20 months.

**Table 1:** Contextual details of participants

Participant number and pseudonym		Age (years)	Stillborn baby pseudonym	Stillborn baby’s gestation	Live baby pseudonym	Live baby age (months)	Partner/Husband pseudonym	Diagnosis history
1	Ruth	35	Emma	41	William	4	Steven	No
2	Sharon	32	Oliver	31	Grace	4	Kevin	Depression
3	Sarah	34	Joseph	34	Jacob	21	Dylan	No
4	Karen	48	Chloe	32	Shauna	48	John	PTSD
5	Isabel	28	Ella (Mia twin sister)	25	Amelia	30	Richard	Depression
6	Defne	30	Ufuk	30	Zeynep	48	Murat	No

### Procedure

Information about the study was given to participants along with the consent form. All participants gave fully informed consent including explicit consent for their quotes to be used for research related publications. After completing a consent form and a demographics questionnaire, four open-ended questions were asked of participants (please tell me about your stillbirth experience; please tell me how you felt during your subsequent pregnancy; describe how you felt when you first saw your subsequent infant (first thoughts, first memories); Please describe your relationship with your child and has there been any change in your relationship with your child from birth to present?).

In addition, they were also requested to provide further explanations about their accounts to clarify some aspects of their shared experiences. Participants of the study were asked to write freely as much as, or as little as, they would like to write about their accounts. No time limit was given to participants. They were free to withdraw from the process at any time. The questions of the study were asked one at a time and further clarifications were requested in order to clarify the individual’s experiences (e.g. if they used metaphors, they were asked if they can explain further what they mean by this). The questions of the interviews were asked over a period of time. In total 6–8 emails were exchanged between the researcher and the participant. For the purpose of the research, participants’ personal and contextual characteristics were taken out and pseudonyms were used throughout, including participants, partners, babies and other children.

Participants were firstly asked about their stillbirth experiences; secondly, about their experiences of the subsequent pregnancy; thirdly their memories of giving birth to their living infant and finally their relationships with their living infants were examined. Each participant completed the email interviews within a time range varying from 8 weeks to 12 weeks. Once the email interviews were completed, debriefing information, including further information and relevant support services, were provided.

## Results

An interpretative phenomenological analysis (IPA) was used in this study [21]. Our analysis focused on how women make sense of their experiences of their stillborn baby and their relationship with their subsequent child, as well as the common ground shred by these women. The process set by Larkin and Thompson [23] was taken as a guide and the following procedure was adhered to during the analysis of participants’ accounts (see Table 2). IPA analysis produced three principal themes: I). ‘Broken Canopy’; II). ‘How This Happened’; and III). ‘Continuing Bonds. Overall 11 subthemes (Table 3) were identified which are illustrated in the main features of the principle themes. The titles and labels of themes contain participants’ own wordings where possible in order to stay close to the participants’ own experiences. Some interpretation and discussion points were also highlighted within the themes’ narrative. Then under the main theme headings all the themes were presented and discussed.

**Table 2:** Steps taken for the IPA

1. Prepared transcripts for analysis (pseudonyms were given, identifying details were taken out and line numbers inserted). (By first author)
2. Free coding followed up by a close, line-by-line analysis, was completed to understand each participant’s concerns and claims. (By first author)
3. Emerging themes were then established for each individual case in conjunction with regular supervisions. (By first & second author)
4. Then, from the researcher’s own understanding of theoretical frameworks in Psychology and from reflections from her own stillbirth experience, an interpretive dialogue was established and this was also highlighted in each transcript. (By first & second author)
5. For each case a narrative overview, summarising emerged themes and the researcher’s own interpretation and speculation for each case, along with the line by line coded transcripts, was established. (By first & second author)
6. All participants’ identified themes were presented side by side in a table for general visual overview. This was then used towards establishing the structure of the main and sub themes. (By first & second author)
7. Then all participants’ experiences were tabulated, this time according to the established structure and presented in a table in which the participants’ contribution was indicated. (By first author)
8. A narrative of women’s experience, evidenced by extracts from participant’s accounts, was then developed in conjunction with the established structure. (By first author)
9. The final analysis and interpretations were also overseen by the 3th and 4th authors, and the overall findings in relation to perinatal loss, attachment and mental health literature were assessed.

**Table 3:** Women’s experience of stillbirth and parenting experiences of subsequent infant

I) Broken Canopy		The number of participants shared the experience
1. It cannot be true – Baby with no heart beat	(Pregnancy with a dead baby; Confronted by a dead baby; Choice and information)	6
2. Questioned Self & others	a. The off script experiences of others	6
	b. Others failure to acknowledge the loss	6
	c. Changed view of self – self is alone	5
3. It cannot be true – Baby with a heart beat	(Consolation prize/Runners’ up prize)	6
4. Surreal Experiences	(Joy and grief; Creating life like other women)	6
5. Anxious parenting	(Unrealistic expectations from self; Creating memories)	5
6. Integrating death in life	Self-growth	4
II) How did this happen?		
1. Why	Is the self the culprit?	6
2. Emotions	Anger and despair	5
III) A Continuing Bond		
1. My baby existed after all	They are brothers/sisters; We are a family; He/she is still my child	6
2. Betrayal		4
3. Longing and need to be in touch		6

### Theme I - Broken canopy ‘Questioned self and the changed view of world –world may not be safe’

One of the participants offered an image of a ‘protective canopy’, a symbol of the assumptions which we may make about the safety of the world. **It reflects a concern, shared by all our participants, about the puncturing of this canopy with the unexpected death of a baby*.*** Respondents described something akin to an **existential crisis**: the realisation - through the deaths of their infants - the ‘safe world’ was actually fragile and were vulnerable. The realisation that anything could happen to the canopy appeared to change their world view. The vulnerability of self and life became the new focus, as it is represented in **Karen’**s extract below:

*I think now that Chloe's death has left me with an almost constant awareness of the fragility of life, how quickly everything can change. Before Chloe died, a headache was just a reason to go to bed earlier, now I worry could it be something more serious. Now when friends are expecting babies, I feel great relief when their babies arrive safely. I don't have that blind expectation that all will be well. I don't trust doctors so much either****(KAREN)***

This main theme, collectively shared by all the participants in various forms, presented itself in 6 sub themes: ‘It cannot be true - baby with no heart beat’; ‘Self and Others’; ‘It cannot be true – baby with a heartbeat’; ‘Surreal Experiences’; ‘Anxious parenting’ and ‘Integration of death in life’. Table 4 provides an overview for this large theme.

**Table 4:** Sub subordinate themes of broken canopy

Sub subordinate theme	Quote	Shared experiences
*1.1 It cannot be true - Baby with no heart beat*	I couldn't breathe. I couldn't speak. Not only did I have to start processing this horrible information, but I had to experience it while still being pregnant…couldn't run. I couldn't fall to the floor. I had to hold up this big pregnant belly (SHARON)	All participants shared their disbelief when they learnt the baby’s heart stopped and their baby was no longer alive, although, they still looked pregnant and they still gave birth to their babies.
*1.2 Questioned Self and Others*	The death of a baby is so "off script". It's just not supposed to happen. And it taps into people's individual fears and discomfort (SARAH)	This ‘off – script’ experience then translated into failure of acknowledgement of such loss by others and the person being isolated from others as a result.
*1.3 It cannot be true - baby with a heart beat*	I know it's an odd observation to make, but I was really astounded by the fact she was breathing (ISABEL)	The realisation of the broken canopy and the heightened awareness in danger and death appeared to leave the women surprised at having given birth to a living, breathing baby after all. Women collectively reported that they questioned their ability to create life and were prepared to face further adverse experiences.
*1.4 Surreal experiences*	I enjoyed seeing Grace on the screen at our many doctors' appointments. Those were the moments I focused on her… But mostly, my thoughts and focus were on letting my hopes and dreams for Oliver go, and learning how I could incorporate his absence into my life (SHARON)	A co-existence appeared to be linked with the surreal experiences and left mothers in a dilemma. All participants reported simultaneously experiencing opposite feelings – joy and grief, were reported by all the participants.
*1.5* Anxious parenting - (Unrealistic expectations from self; creating memories).	This is all after she was born -going through the labour with her was a different thing entirely! I have described the change over from being in labour to having her born as two different worlds -diving off of a cliff only to land in a foreign land (SHARON)	Heightened awareness of the imminent danger along with surreal experiences appears to influence mothers’ relationship with their infants and their parenting experiences.
*1.6* Integrating death in life	In fact, there had always been that fear of driving. It was difficult to imagine myself in traffic jam. But today I drive to work every day (DEFNE)	The awareness of fragility of life and death itself appeared to bring a new authentic way of living. Life and death are not separate entities. Four out of six participants, articulated being able to find new ways of in engaging with life. Their focus appeared to move to the ‘present’ ‘here and now’ as described by Defne.

#### It cannot be true - Baby with no heart beat

The arrival of the baby who died in the womb appeared to bewilder women as the natural process of the pregnancy, and consequently the birth, was completed but without any living baby at the end.

**Women also seemed not to be sure what to expect from labour, especially about meeting the dead baby.** They were puzzled as their deceased babies were no different to a living babies in terms of the way they arrived and the way they appeared.

*She was wrapped in a towel, like any other new born baby and handed to me. She was absolutely minute. Her face was bruised and there was a tiny trickle of blood coming from her nose and mouth. Her eyes were still sealed shut and she had no hair. She was still, clearly, meant to be in my womb. I held her and cried over her for a bit, before handing her to my husband who did the same****(ISABEL)***

**Isabel**, in response to meeting her 17 weeks gestated baby stated that “it was still clearly meant to be in my womb”. This summarised the out-of-place experience and also suggested a realisation that the full term was not complete and baby was too small to live and therefore there was nothing that could be done without the completion of the process.

In addition, half of the participants reported that they still held hopes for meeting a living child at the end of this natural process, regardless of the facts that they were given. **It appeared that the actual realisation of the baby’s death did not happen until they met their baby in the flesh.**

*No-one had told me Chloe would be warm. I think deep inside without telling anyone I felt she was still alive. Later as she lay in the cot beside me I dozed off and when I woke I thought she had moved I screamed. The junior midwife came running in. I didn't tell her why I screamed****(KAREN)***

**Upon meeting their stillborn babies, mothers appeared to instinctively want to take care of them.** Not being able to do so seemed to make mothers anxious, and particularly when they were separated from their babies, they seemed to realise the fact that they were not going to see their babies ever again.

*We were able to hold him and spend some time with him, then they took him …****When the hospital took Oliver away****, I felt empty. I wanted to know where he was going, who was going to take care of him, were they going to be careful even though he wasn't living. He was my child and I felt sick that I would never see him again. For me that was the beginning of my unyielding grief that he was no longer a physical part of me and I couldn't feel him anymore****(SARAH)***

*‘When the hospital took him away’* also suggests the mother’s struggle in accepting her baby’s death. This may suggest belief that he is still alive and also her helplessness. Furthermore, it emerged that women **were not informed about the options available** after their stillbirth experience and because of that **some women missed or almost missed their chances of being able to say goodbye to their baby**. For example, some only found out by chance about the possibility of keeping their baby overnight. While women were recovering from the demanding labour itself, these women were at the very same time also going through the realisation of their baby’s death. **Karen** cited her gratefulness for being able to say goodbye to her baby, even if only by chance.

*We found out on Monday, 3 days later that we didn't have to leave her there so we went and brought her home. It was so lovely to hold her and hug and kiss her and have her home. She was in her coffin in our bedroom until we buried her on Wed. Having Chloe home meant the world to us. Her big sister was able to hold her too and family visited and we felt we had 48hrs to tell her we love her. We never think about the fact we were not told we could take her home. That would be so upsetting. We are just thankful we found out we could bring her home****(KAREN)***

**The importance of keeping a memory of the deceased baby becomes clear to most mothers at a later stage in their journey, as suggested by Ruth.**

*We were fortunate to have a local photographer from a local charity arrived at the hospital and took pictures for us. It seemed awkward at first but we are both very thankful to have these photos as they are the only ones we will have of our darling angel daughter****(RUTH)***

Five out of six participants seemed to acknowledge the importance of being able to take the available opportunities (taking photos, taking the baby home etc.) later on. One of the participants (**Defne)**, however, was strongly encouraged by significant others ‘to move on’- get on with life and hide her feelings and her longing to see her son. This may be one reason why she did not name her baby, had no physical memories of her baby, and relived her experiences on her own secretly.

#### Questioned self and others

**It emerged that the mothers’ stillbirth experiences triggered fears and threatened their own assumed safe world.** Acknowledging this experience perhaps risked acknowledging the possibility of the fragility of someone’s own canopy. Perhaps this was the underlying reason for others’ unavailability for support and validation, and for their suggestions of dismissive strategies to the bereaved mothers.

Collectively all participants shared their **need to be recognised and acknowledged by significant others following their experience of loss.** However, validation of their feelings from others did not appear to be available or else was limited.

*Only one of my friends said cry Defne. No matter what I will say will lessen your pain, but express your feelings to me- offered me a shoulder to cry on. I cried a lot that day, only to her… How well she understood my only need to be able to cry****(DEFNE)***

*Every now and then someone either a family member, friend or someone handling the burial arrangements would make a hurtful comment such as "Don't worry, you'll have another baby." or "Are you sure you felt fine? You didn't feel like anything was wrong?" or "It was God's will." None of this was helpful, because a) I don't want another baby. I want this one; b) if I didn't feel fine, I would certainly have rushed to the doctor or hospital!; and c) what little faith we had we were now questioning ****(SARAH)***

Women appeared to be **being isolated and alone in their experiences in this unknown new world.**

*I think it was tough, dealing with depression to be honest and my friends, the one or two I have in [Place 1], rarely showed up or text me, so they were little help sadly. My husband worked 60 hour weeks at the time, so when I did see him, he was exhausted himself****(ISABEL)***

**The majority of mothers with their unmet needs of support appeared to be encouraged to keep their sorrow within as they felt lonely and isolated in their experiences.** There appears to be a changed view of self and others and feelings of isolation and loneliness.

*It is wonderful to go to school with her everyday but I still cry when I think of my son but nobody knows it ****(DEFNE)***

#### It cannot be true – baby with a heart beat

Women appeared to question **their ability to create, with the arrival of the dead infant.** However, until the arrival of the subsequent living infant, the concern that they may not be able to create or bring life was a strong possibility within the shattered unsafe canopy. Therefore, it appeared to be hard for the women to believe that they could have a living baby after all.

*This may sound morbid, but I felt disbelief that I had actually given birth to a healthy child! ****(SARAH)***

**Women, seemed to try to separate the two different, but co existing, infants’ places throughout their journey.** This breathing baby was not a substitute or consolation prize. Mothers refused to think that they were substituting their children with each other, as represented in Karen’s account.

*Other people seem to see Shauna as some kind of ‘consolation prize’ for Chloe's death. I find this so untrue and offensive. Giving birth to Shauna safely and rearing her did not heal my grief over Chloe. What it did do was give me a pressing reason to get up every morning. One child does not replace another. Each of my daughters has their own special place in my heart****(KAREN)***

#### Surreal experiences

**Co-existence on the other hand appeared to allow mothers to define each child.** Sometimes the dead baby defined the existence of the living baby and sometimes the living baby defined the existence of the dead baby throughout the mothers’ journey, as described by Sarah’s extracts as shared in the co-existence:

*Often I felt I had to act like I was always happy and grateful in front of everyone else for their own relief and happiness about expecting Jacob. Mind you, I was thrilled to be expecting him, but that coincided with the fact that I was still grieving. The guilt didn't last long, because we came to see Jacob as a sign from Joseph that we should love another child as well. Perhaps that sounds a bit esoteric, but we believe that****(SARAH)***

#### Anxious parenting - (Unrealistic expectations from self; creating memories)

When **Sharon** was asked to describe her relationship with her living subsequent infant, she described the loss and despair as a ‘cliff’, and that giving birth to a living child was like ‘diving off from this cliff to land of an unknown – parenting’. This analogy sums up the other women’s experiences of how their parenting was influenced and shaped by their previous loss. Life and loss coexisted once again.

**Joy coupled with grief, and the shattered ‘safe’ world seemed together** to catalyse the women’s constant worry of their living offspring’s welfare. Mothers appear not to focus ‘here and now’, but rather their focus is either in the past or in their future worries.

*There was so much worry. I had dreamed before she was born, of us being so relaxed and enjoying her baby time. I think now that my expectations were too high. But the worry for having Shauna did not turn out how I expected. I was surprised when one doctor commented that he couldn't understand why we worried so much about her. After Chloe died so suddenly, I felt it made perfect sense that we would be worried that something bad would happen to Shauna ****(KAREN)***

Furthermore, half of the participants appeared to be engaged in protective mothering activities, sometimes involving unrealistic expectations of self in order to protect the infant in an **unsafe** world.

*My feeling toward my ‘importance’ to Daniel relate to my ability to provide for him in a way that no one else can. I really wanted to be able to breast feed him for at least 6 months. When this didn't happen, I felt less of a woman and almost helpless ****(RUTH)***

The constant awareness of the fragility of life and the emphasis on ‘past’ and ‘future’ motivated most of the mothers to anxiously collect mementos of their subsequent children. And this time, mementos are plentiful, unlike the first time. Now that they know how precious keepsakes can be, collecting them may be a way for mothers to protect themselves – if this subsequent baby should die too, they will have more mementoes to keep and treasure, which would bring comfort as they grieve. This can make the risk of another loss more bearable to contemplate.

*With Grace, if there was something that felt right for her, I bought it with the understanding that it is hers whether she ever used it or not. In that way, I was creating physical memories of her if we lost her****(SHARON)***

#### Integrating death in life

**The awareness of fragility of life and death itself appeared to bring a new authentic way of living.** Life and death are not separate entities. Four out of six participants, articulated being able to find new ways of in engaging with life. Their focus appeared to move to the ‘present’ ‘here and now’ as described by Sharon.

*Something beautiful that I experienced being pregnant after having a baby born still is that I treasured each moment that she was alive in me. Most people go through pregnancy anticipating the next steps - birth and life. Never having gotten to those steps with my son, I was able to build a relationship with my daughter in a unique way in utero. I was getting to know her and think about her in the moment rather than dreaming about the future****(SHARON)***

Mothers appeared to be anxious about being able to protect their children in their shattered new world. They then tried to restore the broken canopy at any cost including sacrificing themselves via unrealistic expectations of the self. Heightened anxiety appeared to be a new focus in their relationship with their infant. However some women moved beyond their awareness of death and danger, and integrated the death and danger into their life, existence. Life and death together defined their existence**.** Gathering memories also requires that a mother soak up the present moment, which is another way to express an enhanced appreciation for this new baby’s life.

In summary, it appeared that the realisation of the ‘vulnerability of self’ spurred participants into an appraisal process where their own abilities and others’ availability to them were assessed. The view of an assumed - safe world changed and women appeared to feel isolated in their experiences. When women then turned to others for support they were urged to move on – get on with life. This meant to mothers that their experiences were dismissed and rejected and this was deeply upsetting for women. Still, even as each mother turned toward her subsequent baby, she did not abandon her devotion to her deceased baby. The two babies appeared to co-exist with each other, as separate individuals, and the mother’s experience of each coloured her experience of the other. For example, the arrival of a new baby could not be embraced by mothers as a completely new experience, as it was coloured by grief for the baby born still. The mother’s experience of losing and grieving for her deceased baby rendered bittersweet her experience of welcoming and parenting the new, healthy baby. In kind, as the mother’s emotional investment turns to the desire to create and nurture a new life. At the same time she worries she is betraying her deceased infant. This contradictory duality is articulated in powerful, simultaneously experienced opposing emotions like joy and grief, fruition and guilt.

### Theme II – How did this happen

Women collectively asked the question ‘Why’ in their accounts. One participant, Ruth described losing her baby as tragedy. This suggested extreme sorrow, as a consequence of a tragic flaw, however there was a meaningful ending.

*When I am reminded of my daughter's tragedy I think to myself how lucky I am to have known her at all. I used the knowledge of her situation and took that forward with me during my pregnancy with William… Life is so precious and too many people take the ability to create life for granted (****RUTH)***

Other women articulated answers for their quest in searching for answers as to why this had happened, and their account did not have self-blame as shared by Sarah and discussed under the co-existence subtheme.

#### Why - Am I the culprit?

It appears that when there were no meaningful answers to the question of why they were chosen to live without their children, most of the women (Five out of six participants) expressed anger in the form of self-blame towards self.

*So, basically, we were left with absolutely no answers. I think that has been the hardest part in our process. Doctors tell us they don't like to have answers because it's less likely to recur. We like that. But it doesn't help in our understanding of what happened to our little boy. And it certainly adds to my anxiety that maybe it was something I did (****SHARON)***

#### Emotions - anger and despair

Anger was also articulated by almost all participants, except Ruth, toward various significant others including family, friends, hospital staff, God and the baby.

*Everybody said if this happened later it would have been worse, what happened was better than what would have happened if this child born with disabilities. This made sense but it did not make me feel better. Even it made me angry****(DEFNE)***

Isabel, in response to a consultant’s dismissive statement for her constant worry during her subsequent pregnancy, stated that

*I could have chucked a chair at his head. I pointed out to him, rather curtly, that I had buried two children and that I didn't plan on doing it again and if he'd been through what my husband and I had been through, he wouldn't be asking such a dumb-ass question****(ISABEL)***

Anger was also expressed towards God, and the baby, in participants’ Sharon’s and Karen’s accounts.

*I could not touch him. When asked prior to his birth, I had told everyone I was going to hold him. But when he came out I felt differently. All I kept thinking was, "That's not him" I knew the real essence, the true being who had been my little boy, was not in that body. My baby was gone. I said I had held him for eight months. I wasn't going to hold him when he wasn't there****(SHARON)***

*I tried going to Church but gave that up quite soon as I was so angry with God****(KAREN)***

Women collectively also talked about their despair and helplessness in the situation that they were in while articulating their realisation that there is nothing they can do or undo to change the circumstances; this is represented by Ruth’s extract.

*I spent the first few days just completely numb, like a robot, coordinating and planning her funeral services. I felt the need to make sure she received the best she could, since there wasn't anything else I could do for her****(RUTH)***

In summary, sooner or later, the arrival of a sudden ‘end before a beginning’ appears to come as a shock to all women. Then they appeared to continue to question the self and others, while looking for the reasons why they had to go through such experiences. All participants expressed despair, while anger towards self and others was shared by most of the participants (Five out of six participants).

### Theme III - A continuing bond

A final theme emerged around the enduring relationship with the deceased child, which was articulated by all the participants. As discussed in the summary of the “Broken Canopy” theme, the mother’s enduring bond was also observed in the co-existence of her deceased and living infant. This was also observed in the co-existing relationship between deceased and living infants. Three subthemes emerged under this theme as follows:

#### My baby existed after all

The death of an infant before birth giving rise to hardly any memories seems to complicate the natural bereavement process. Women are faced with a baby’s death before fully realising this baby’s existence. It appears that this realisation becomes clearer only with the arrival of the new baby. This collectively shared experience was articulated in Sharon’s excerpt.

*There is the real child, and the one we have created in our minds. Understanding this was a beautiful and helpful thing for my relationship with Oliver. It made part of him still exist for me. Accepting this allowed me to continue to know him as my child. I just had to come to terms with the fact that I would never get to see who he was as compared with my creations ****(SHARON)***

#### Betrayal

As mentioned under the “Broken Canopy” theme, the majority of participants (4 out of 6), felt that they have betrayed the deceased infant when they became pregnant again and subsequently gave birth to a living infant. Women’s natural desire to have children appeared to contradict the desire to have children appeared to contradict the desire to feel connected to the baby who died, as illustrated by Sarah.

*It was very complicated. I felt terribly guilty, as if we were already forgetting Joseph. I'm sure others judged the fact that we conceived right away, but our doctor recommended it and I was already 33. Even when I discovered the positive pregnancy test, I remember calling Dylan and just feeling scared and nervous. It was difficult to enjoy the pregnancy and all the joys of expecting--first kicks, ultrasound photos, etc. We were constantly fearing for the baby's life ****(SARAH)***

It also appears that the majority of women at a later stage in their journey reflected on how they dealt with the process and regretted the missed opportunities afterwards, as articulated by Sharon.

*She presented me with a card with the footprints in and photos of her. I was very grateful for her doing that, although I think back now and wish I'd have done it myself. I was her Mum after all ****(SHARON)***

#### Longing and need to acknowledge a continuing bond

Women expressed a longing for their infants and the need to stay connected somehow.

*‘My husband and I felt incredibly lonely in the sense that we had these empty, aching arms that should be more than filled with two babies****(ISABEL)***

Women chose different ways to be in touch with their baby and their memories:

*Ok, keep the questions coming. I am glad to be purging all of this. Sometimes I go weeks without talking about Joseph ****(SARAH)***

And some stayed in touch with their infant via involving themselves in activities in their child’s memory, supporting families going through similar experiences or taking part in research in the area of stillbirth experiences.

*I just love to talk about my daughter; it helps to ‘keep her alive’ in my heart****(RUTH)***

One of the participants said that although their infant is not living anymore, the child is still part of their family, including the subsequent children. This loss is also the whole family’s loss including the subsequent children.

*I walk around thinking I should have two sons on either side of me. My husband says we are blessed with our son Jacob because we lost our first son Joseph, but I still feel that they are brothers who should be together right now, playing, getting into trouble, getting ready to start nursery school, etc. I think of myself as a mom to two boys, but no one else sees me that way****(SARAH)***

As discussed in “Broken Canopy” it appears that the co-existence of the dead and living babies enables the mother to be connected with her deceased infant for whom she still feels a longing and devotion. **Isabel** reported explicitly how she connected with her infants via sensory experiences with her living infant.

*Coped with the guilt of devoting all my time and attention to Amelia by doing certain things. Probably sounds weird, but there are times when I can 'smell' them. All babies have a particular scent and so did Ella before she died. From time to time, I can smell her and I always say hello to both her and Mia****(ISABEL)***

In summary, mothers appeared to be initially occupied with making sense of their baby’s death. The recognition of the baby’s existence is something articulated in all mothers’ accounts at a later stage. At the same time mothers expressed longing for the baby who died and the need to acknowledge their maternal bond. Betrayal of the deceased infant was also expressed when the mother experienced joy. They reflected on their missed opportunities, such as spending more time with and holding this baby, and keeping memories alive so they can continue to feel connected with this child.

## Discussion

Women’s accounts revealed that the experience of stillbirth is a process where women **re-visit** the experience and reflect their experiences **throughout other life events such as the arrival of a new baby.** The experience of stillbirth appears to **influence the relationship with the subsequent infant and parenting.** Further discussion and interpretation points, for the three main principle themes follow:

### Broken canopy

Women collectively appeared to question their sense of mastery in the world, and the foundations upon which they build their lives following their stillbirth experiences. It is plausible that an existential crisis as discussed by Yalom [24] appeared to be evident in participants’ accounts, particularly, in debating thoughts of existence vs nonexistence and the fragility of life. Similarly Janoff – Bulman [25] discusses how the death of a loved one shatters individual’s world view and core assumptions about self. Women’s increased awareness of death and their questioned ability to be able to cope appeared to be translated into **constant awareness and anxiety in** the **subsequent parenting experience**. This finding is in line with the emerging literature for parenting following perinatal loss [18, 26]. Yet some women reported an integration of the baby’s death into **their lives, a self-growth** following their trauma experience, as discussed by Yalom [24], Linley and Joseph [27], and Davis and Nolen-Hoeksema [28]. Furthermore, this authentic, enriched perception appeared to enhance the **mothers’ relationships with their subsequent children. This finding is similar to** the findings of a recent qualitative self - growth study following stillbirth [29] and the findings of Cacciatore [30] (2010) and Lichtenthal and her colleagues [31] (2010) following the death of an infant. The current study extends this understanding to the subsequent parenting experiences of mothers and supports the emerging findings in relation to the influence of self-growth on parenting [32] (O’Leary & Warland, 2012).

From an existential point of view [24] women were faced with the possibility of their inability to be immortal by generating life and so had the urgency to try for other babies. At the same time women also needed to grieve and continue to feel connected with their stillborn babies and their memories. This desire to have more children in order to generate life, seemed to have left women with the dilemma of worrying about betraying their deceased infant [30, 33].

The fact that mothers questioned both themselves and others in response to such an existential threat can also be examined from the perspective of attachment theory [34]. Bowlby suggests that attachment behaviour is activated under a threat and individuals then engage in support-seeking behaviour. People with insecure attachment, who had inconsistent or lack of security-providing-interactions with attachment figures, are expected to have doubts about the effectiveness of available support, and will use other secondary strategies (such as, the use of deactivating strategies to idealise and normalise relationships, no or lack of memory in relation to early care experiences [35], or self blame [36]). The lack of validation of feelings and rejection by others may is an issue for individuals with insecure attachment style and could lead to self-worthlessness and self-blame. All participants collectively wanted their experiences to be acknowledged. Stillbirth, such an ‘off-script’ experience, appears to threaten other people’s own assumption of a safe world, making them less likely to validate the mothers’ experiences and feelings. This in itself appears to isolate women in their bereavement process and forces them to hide or deny their feelings. Mothers found it hard to deal with others’ dismissive approach (e.g. suggesting ‘moving on’ or reminding women to be grateful for their living baby). This rejection also appeared to cause one woman to criticise herself. For example, this participant stopped seeking medical and social help while going through grief, depression, and the demanding needs of caring for her subsequent newborn baby. Being critical of oneself and a lack of social support are both identified risk factors for prolonged grief or delayed grief reactions. Acceptance, however from the wider community helped women to embrace their experience. These findings concur with current research on the importance of social support [37–40]. In addition, the lack of validation of feelings and rejection by others appears to confirm the prior rejection of caregivers and results in the mother’s experience of disenfranchised grief as discussed by Nichols [38].

What was also striking in the mothers’ accounts was the co-existence of contradictory powerful feelings, described by one of the mothers as ‘surreal experiences’. Women reported experiencing joy and at the very same time disbelief, when they gave birth to a living, breathing baby. The joy was also coupled with their grief and longing for the dead infant. The co-existence between the dead baby and subsequent living baby was also reported during the subsequent pregnancy from conception to birth and was even present in mothers’ parenting experiences of the subsequent baby. Mothers appeared to define their subsequent baby’s existence according to their bereavement experiences and they seemed to be able to process the existence of the stillborn baby, who they had never seen alive, via their interaction with their living infant. This co-existence also enabled women to feel a continuing bond with the longed-for infant. However, the women were also aware of each infant’s individual place and existence. Although they acknowledged that one of their infants was not alive, they fought for their infants’ separate places, especially when the outside world appeared to dismiss or ignore this independent existence (‘they are like brothers’; ‘second child is not a consolation prize’).

In addition, it can be speculated that the co - existence of the two infants also helped women in their grieving process, however, this hypothesis needs further investigation in future studies that include controls of women who do not have any living children. The study’s findings regarding co-existence does extend the current “replacement child” and “vulnerable child” debate as discussed by Turton and colleagues [17], while bringing attention to the connection between the deceased and living infant and in some ways, the co-existence being therapeutic for the mother.

### How this happened

Whilst mothers were facing the new existence of a subsequent child, they all questioned their ability to cope and others’ availability for them during their journey. This inevitably gave rise to the question ‘why’, and in response, women expressed anger towards self and others, including significant others. Anger towards self, and others were reported by almost all participants. Questioning of the self and self-blame as part of the anger process was also observed, similar to the findings of another qualitative study [31]. Only one of the participants did not express anger or self-blame towards self and others. This perhaps was due to the way in which she conceptualised the loss, and integrated the death into her life, or the way in which her experience was also acknowledged by her close community, unlike other participants’ experiences. Perhaps the acceptance and validation of their experiences and their feelings at an earlier stage contributed to the person’s own acceptance of the situation without turning the woman’s anger towards herself or others. Further research focusing around the need of validation and acceptance of mothers’ experiences may expand this speculative point. However, it is evident both in this study, and other similar studies [41–43] that support from significant others was sought after stillbirth and needed for the experiences and feelings to be validated.

### A continuing bond

Another overarching theme was about continuing the bond with the dead infant. As discussed by Klass and colleagues [44] the need to be in touch with, and longing for, their deceased infant was shared by all the participants. The adaptive value of this continuing bond has been discussed in the literature [45] and this was evident in the accounts of mothers of the current study. The connection with the baby was sometimes achieved via a living baby, by holding on to their few memories, experiencing co-existence, engaging in activities like research, or supporting other families. This theme also emerged in another study where women’s accounts of stillbirth experiences were examined in relation to self-growth [29].

According to attachment theory, the need for a continuing bond can be an indication of failure to integrate the death of a loved one, and individuals can be classified as having an unresolved state of mind with loss. Some mothers in their accounts refer to their deceased infant as ‘gone’, which could imply “just left, not dead” or “had been left at the hospital”. Using this euphemism could suggest a disorganised belief in relation to loss and unorganised state of mind. However, the unresolved category should only be given when there is a disorganisation or disorientation in discourse or reasoning by the individual during the discussion of traumatic events (e.g. loss, abuse) [46]. Although “unresolved griefs” was not explored in this study it is important to understand this dynamic in order understand and appreciate the adaptive value of continuing bonds.

In the current literature, there are contradictory findings in terms of the adaptive effects of continuing psychological and emotional bonds with the deceased loved ones. Klass and Walter [47], Field [48] and more recently, Field and Filanosky [49] identify continuing bonds as either internal or external continuing bonds (CB). Their analysis, inspired by attachment theory, revealed that external CBs (illusions and hallucinations) were positively correlated with guilt and feelings of responsibility for the death, whereas internalized CBs (use of deceased as an autonomy - fostering secure base) were negatively associated with identified risk factors as well as uniquely associated to personal growth. In the current study it appears that the grief process becomes complicated in the stillbirth experience, as death occurs before life outside the womb and there are so few memories to hold dear, which engenders a feeling as if the baby never existed. Therefore, it stands to reason that supporting mothers in being able to acknowledge the baby’s life and death will help them accept their infant’s existence while knowing they are not living. Perhaps this dilemma is one of the reasons for worsening mental health problems in these mothers, such as continuing depression and PTSD [17, 50]. It can be speculated that the issue in the current debate about the link between PTSD and seeing and holding the deceased baby lies in the existence of externalised continuing bonds between mothers and their deceased infant as discussed by Field and Flonosky [49]. Further research is required to investigate this possible relationship.

### Importance of findings

The findings of this study provide **an insight into the stillbirth experience** of mothers and its meaning to them with **an existential focus.** It highlights the dilemmas and difficult decisions that women face in their experiences. It also provides **evidence for the importance of a continuing maternal bond, how the stillbirth experience influences the mother’s subsequent pregnancy and parenting.**

This study reveals that the **mothers’ struggles to accept the existence of her baby while being aware of the non-existence of her baby, as she has no shared or past memories other than those of the pregnancy and birth.** It can be speculated that the lack of memories and opportunities to nurture her baby perhaps then complicates the grief process [51, 52] and may keep the mother in denial or extended the recovery period where symptoms of depression and PTSD.

Every mother in this study saw her baby, although not everyone chose to hold her baby. None of the mothers regretted seeing her baby. Only one research group suggests a link between PTSD and seeing and holding a deceased baby [15]. However, in the current study, it seems that **meeting with the dead baby** actually **was a crucial point at which women started processing their grief.** Only from this point onward was there a full acknowledgment of their baby’s death, unlike the experience of pregnancy with the dead baby and giving birth to the dead baby. These findings are in parallel with the recent related findings and advice [5–8] that mothers benefit from health care practitioners assuming they feel a maternal bond and then supporting them around spending time with their babies.

Although **seeing the deceased baby seems to facilitate the grief process, the established strategies of each individual were important while they were dealing with the emotional aftermath of meeting their dead baby.** For example, a mother with dismissive strategies or mothers with avoidant attachment styles, may find it difficult to process such direct contact. Although this is a speculative point it is, however, important to note the importance of **individual differences in dealing with stressful situations** when providing efficient guidance in the management of stillbirth. Therefore more research should be carried out to understand individual differences in dealing with stillbirth experience and this should then inform the relevant guidance It is also important to note that participants did not receive clear information about their options for spending time with their stillborn babies. This could be because of a hesitant attitude of the staff due to the current, mixed guidance. **Therefore a clear and unified guidance is essential for better stillbirth bereavement care** [53].

Furthermore, mothers’ awareness of danger and heightened anxiety, along with their unmet support needs, were present during their subsequent pregnancy and their parenting of a subsequent infant. These findings are in line with the available literature [12, 54–56]. Anxious parents can become controlling and critical, may experience difficulty in bonding, and subsequent infant attachment can be disorganised [15]. This has further implications for the subsequent infants’ adult life, including the possibility of anxiety and depression disorders [57].

### Limitations

Due to the nature of the qualitative design, the sample of the study is small. Therefore, generalisations of the findings are limited. In addition the women who participated in the study were recruited via web based social networks thus it is only representative of mothers’ who have actively sought more public venues to reflect and share their stories and remain in touch with their infant.

## Conclusions

First of all, the findings of this study inform the professional practice for pre and post-care of mothers who experienced stillbirth. It provides a better understanding of mothers because it explains the meaning to the mother of a stillbirth. Particularly relevant for psychological support services is that emphasis should be placed on the acceptance of the dead baby and co-existing experiences (e.g. joy & grief; betrayal & fruition). Including the deceased child in the family and its narrative may also allow the mother to integrate her stillborn baby her into her life and allow her to realise that her baby existed but is no longer living. This may allow women to grieve, honour her maternal bond, and stay in touch with their baby’s memory. Mothers’ need for a continuing bond should also be recognised and the unmet validation needs of women should be part of the psychological support process. Issues around anxious parenting should be expressed and addressed appropriately, taking into account individual needs. The findings from this study could also inform public health authorities regarding the need for awareness of stillbirth and a better stillbirth bereavement care (e.g. available information, support in difficult decisions) and that individual differences in response should be taken into consideration. Particular attention should be given to the isolation that women experience due to their ‘off-script’ experiences and disenfranchised grief. The findings of this study also had personal implications in that the researcher had a chance to reflect on her own experiences and remain in touch with her own experiences.
